# Supplementary material for: Paralog dependency indirectly affects the robustness of human cells
Source: Mol Syst Biol. 2019 Sep 24;15(9):e8871. doi: 10.15252/msb.20198871 (PMC6757259; doi:10.15252/msb.20198871)
Supplement: Supplementary file 1 — Appendix [file MSB-15-e8871-s001.pdf]

Appendix

Appendix tables

Page

Appendix Table S1: Number of homomeric (P1P1 or P2P2) and heteromeric (P1P2) paralogs. 2

Appendix figures

Page

Appendix Fig S1: Correlation between CS values across datasets. 5

Appendix Fig S2: Effect of gene LOF for singletons and paralogous genes across cell lines. 6

Appendix Fig S3: The LOFs of paralogous heteromers, defined by 'direct PPI's only, are relatively more deleterious than the LOFs of non-heteromers. 7

Appendix Fig S4: The LOFs of paralogous heteromers, are relatively more deleterious than the LOFs of non-heteromers, across most of the cell lines in the CS datasets. 9

Appendix Fig S5: Paralogs that form heteromers tend to be more deleterious upon LOF than other paralogs, largely independently from the age of paralogs. 10

Appendix Fig S6: Association between the GO gene sets of paralogs, their probability of heteromerization and the effect of gene LOF on cell proliferation, in case of the heteromers defined by the 'direct PPI' only. 11

Appendix Fig S7: Correlations between the effect of LOF of a gene on cell proliferation, mRNA expression and number of protein-protein interaction partners. 13

Appendix Fig S8: Relationship between the effect of LOF of a gene on cell proliferation, mRNA expression and number of protein-protein interaction partners. Related to Fig 4. 14

Appendix Fig S9: Feature importance (shown on the y axis) determined through classification models (shown on the x axis), for 4 CS datasets. 17

Appendix Fig S10: Dependence of the robustness of paralogs (shown on y-axis in terms of CS values) on mRNA expression (y-axis), across 4 CS datasets. Related to Fig EV3E. 18

Appendix Fig S11: The most expressed paralog (P1) of a pair is more likely to be 19

deleterious than the least expressed (P2), across 374 cell lines.

Appendix Fig S12: Relationship between the asymmetry of expression and relative deleteriousness of paralog for the CS2 dataset. 20

Appendix Fig S13: Relationships between asymmetry of the mRNA expression and difference in CS values. Related to Fig 6. 21

Appendix Fig S14: Relationships between the asymmetry of the mRNA expression and the difference in CS values is shown for representative heteromeric and non-heteromeric pairs of paralogs. 22

Appendix Fig S15: Structures of the representative heteromeric paralogs showing the number of interface residues. 23

Appendix tables

Appendix Table S1: Number of homomeric (P1P1 or P2P2) and heteromeric (P1P2) paralogs.

PPI type	gene1 homomer	gene2 homomer	heteromer	# of paralogs
all BioGRID				2231
all BioGRID			P1P2	145
all BioGRID		P2P2		248
all BioGRID		P2P2	P1P2	40
all BioGRID	P1P1			217
all BioGRID	P1P1		P1P2	58
all BioGRID	P1P1	P2P2		133
all BioGRID	P1P1	P2P2	P1P2	60
all IntAct				2570
all IntAct			P1P2	42
all IntAct		P2P2		189
all IntAct		P2P2	P1P2	6
all IntAct	P1P1			192
all IntAct	P1P1		P1P2	23
all IntAct	P1P1	P2P2		81
all IntAct	P1P1	P2P2	P1P2	29
direct BioGRID				2704
direct BioGRID			P1P2	11
direct BioGRID		P2P2		169
direct BioGRID		P2P2	P1P2	3
direct BioGRID	P1P1			152
direct BioGRID	P1P1		P1P2	7
direct BioGRID	P1P1	P2P2		65
direct BioGRID	P1P1	P2P2	P1P2	21
direct IntAct				2718
direct IntAct			P1P2	9
direct IntAct		P2P2		156
direct IntAct		P2P2	P1P2	3
direct IntAct	P1P1			172
direct IntAct	P1P1		P1P2	8

direct IntAct	P1P1	P2P2		49
direct IntAct	P1P1	P2P2	P1P2	17

Appendix figures

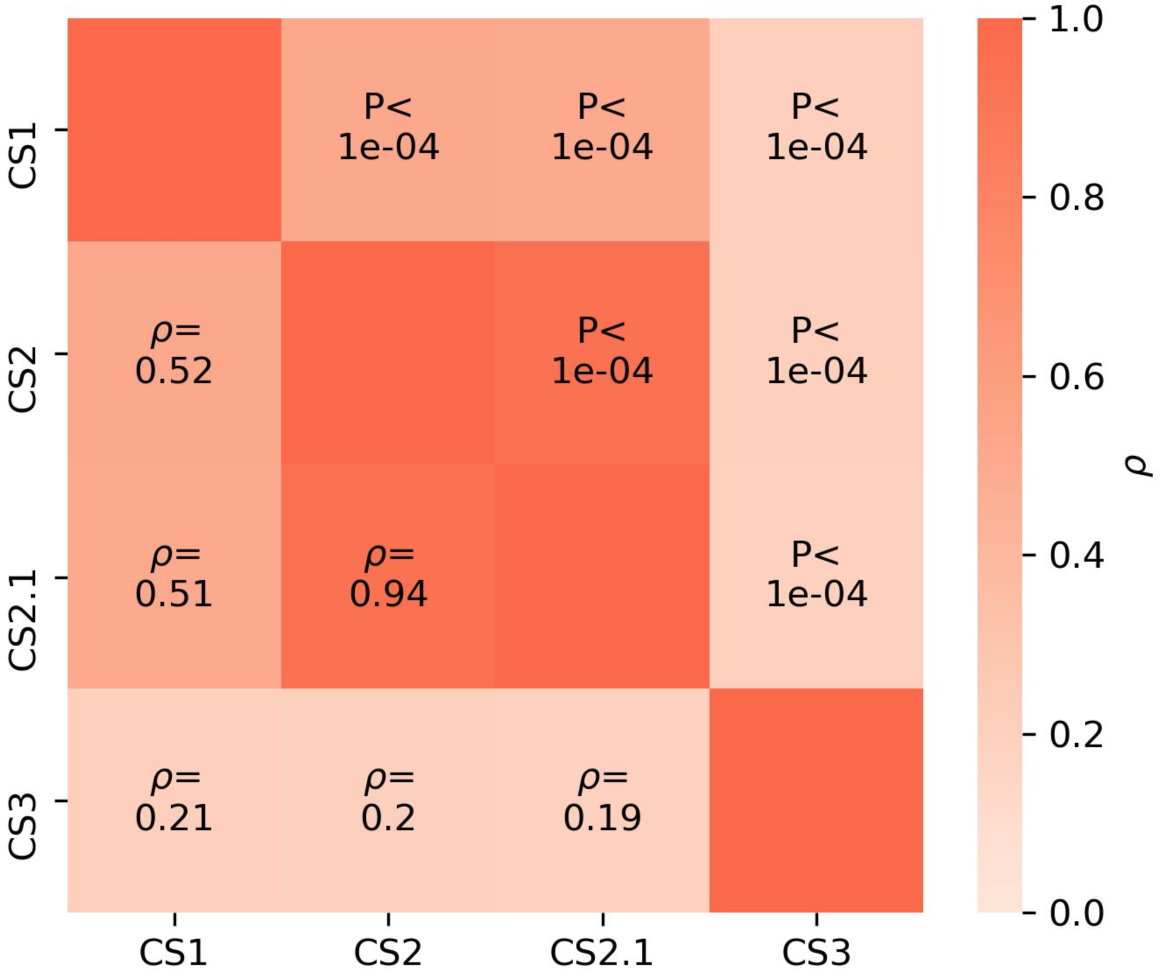

Appendix Fig S1: Correlation between CS values across datasets.
Pairwise Spearman correlation coefficients (ρ) between CS values for the CRISPR screen datasets used in this study. Associated P-values are shown on the heatmap above the diagonal. The most significant correlation is for CS2 and CS2.1, which are CS values derived from the same experiments but with different sets of corrections. The significant correlation reflects that the corrections do not impact the relative ranking of genes.

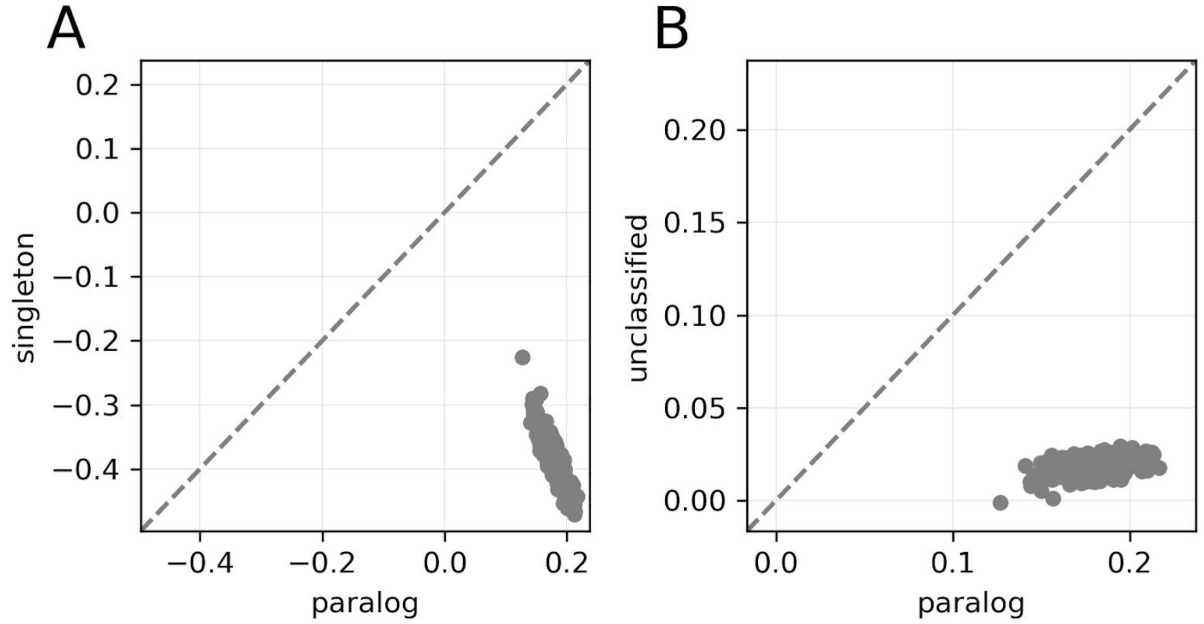

Appendix Fig S2: Effect of gene LOF for singletons and paralogous genes across cell lines.

Unclassified genes are genes that are not in the paralog datasets but that were not identified as singletons in the stringent identification of singletons. Each point represents the mean CS for a class in an individual cell line (450 cell lines from CS2 dataset).

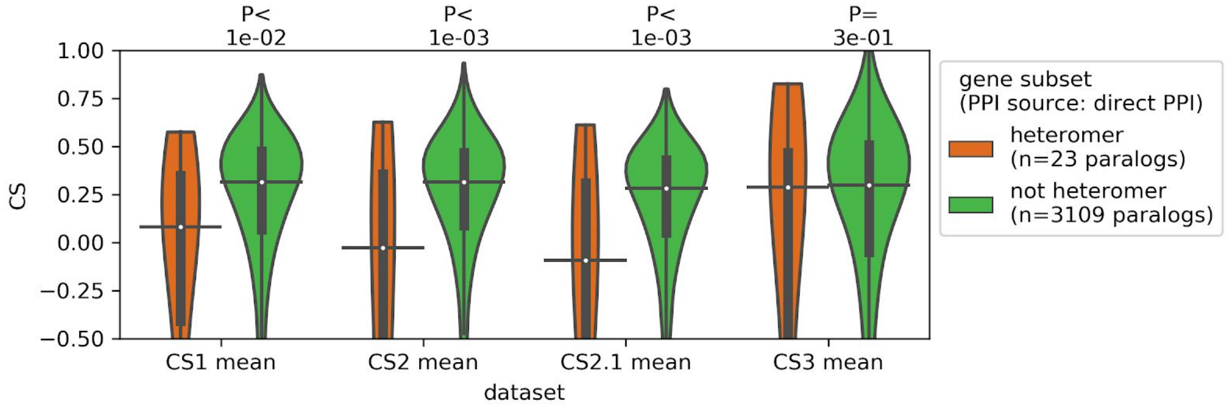

Appendix Fig S3: The LOFs of paralogous heteromers, defined by 'direct PPI's only, are relatively more deleterious than the LOFs of non-heteromers.

Similar analysis as that of Fig 2A.

LOF data derived from genome-wide CRISPR-Cas9 screening experiments. The effect of LOF is estimated by the depletion of gRNAs during the experiment, which reflects the deleteriousness of LOF on cell proliferation. The extent of depletion is measured as a CRISPR-score (CS). Relatively lower CS indicate relative more deleteriousness. CS values across cell lines from three biologically independent datasets — CS1 (Wang et al. 2015), CS2/CS2.1 (Meyers et al. 2017; DepMap 2018) and CS3 (Shifrut et al. 2018) are shown.

P-values from two-sided Mann-Whitney U tests are shown. On the violin plots, the medians of the distributions are by a horizontal black line and quartiles are indicated by a vertical thick black line. For clarity, the upper and lower tails of the distributions are not shown.

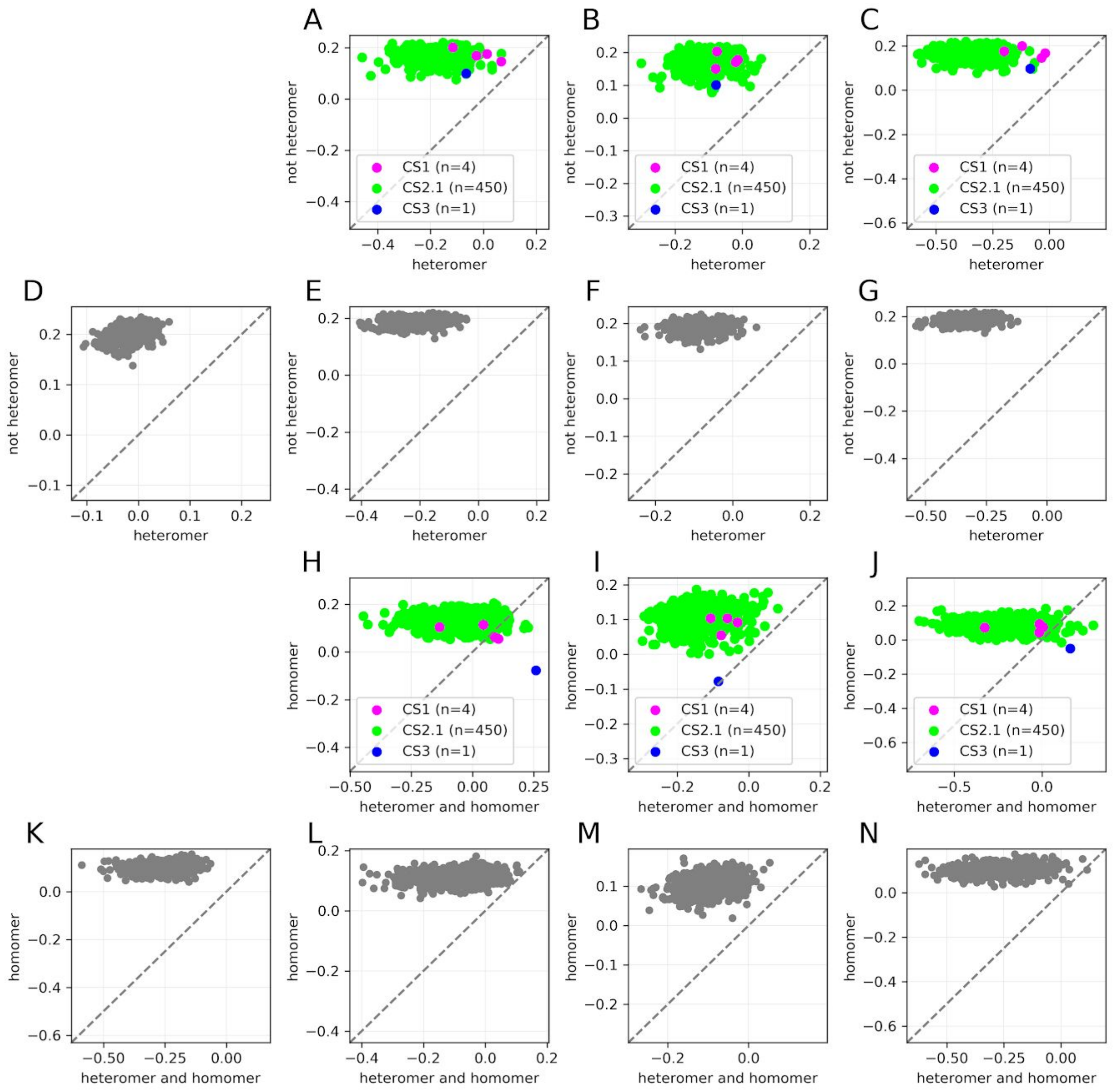

Appendix Fig S4: The LOFs of paralogous heteromers, are relatively more deleterious than the LOFs of non-heteromers, across most of the cell lines in the CS datasets.

Similar analysis as that of Fig 2B and 2C, but with ‘direct PPI’s and CS2 datasets.

The 1st and 2nd rows show the comparison between heteromers and non-heteromers, while the 2nd and 3rd rows show the comparison between heteromers that form homomers and homomers only. The 1st and and 2nd column show to the subsets of paralogs identified from BioGRID, while 3rd and 4th column show the subsets of paralogs identified from the IntAct. Analysis with the CS1, CS2.1 and CS3 is shown in the 1st and 3rd rows. Analysis with the CS2 is shown in 2nd and 4th rows. Analysis with subsets of paralogs (heteromers and homomers) defined by ‘all PPI’s is shown in the 1st and 3rd column while that with subsets of paralogs defined by ‘direct PPI’s is shown in 2nd and 4th column.

In all the cases, the mean CS values per cell line are well separated by the diagonal, indicating that the effects are systematic and independent of cell line.

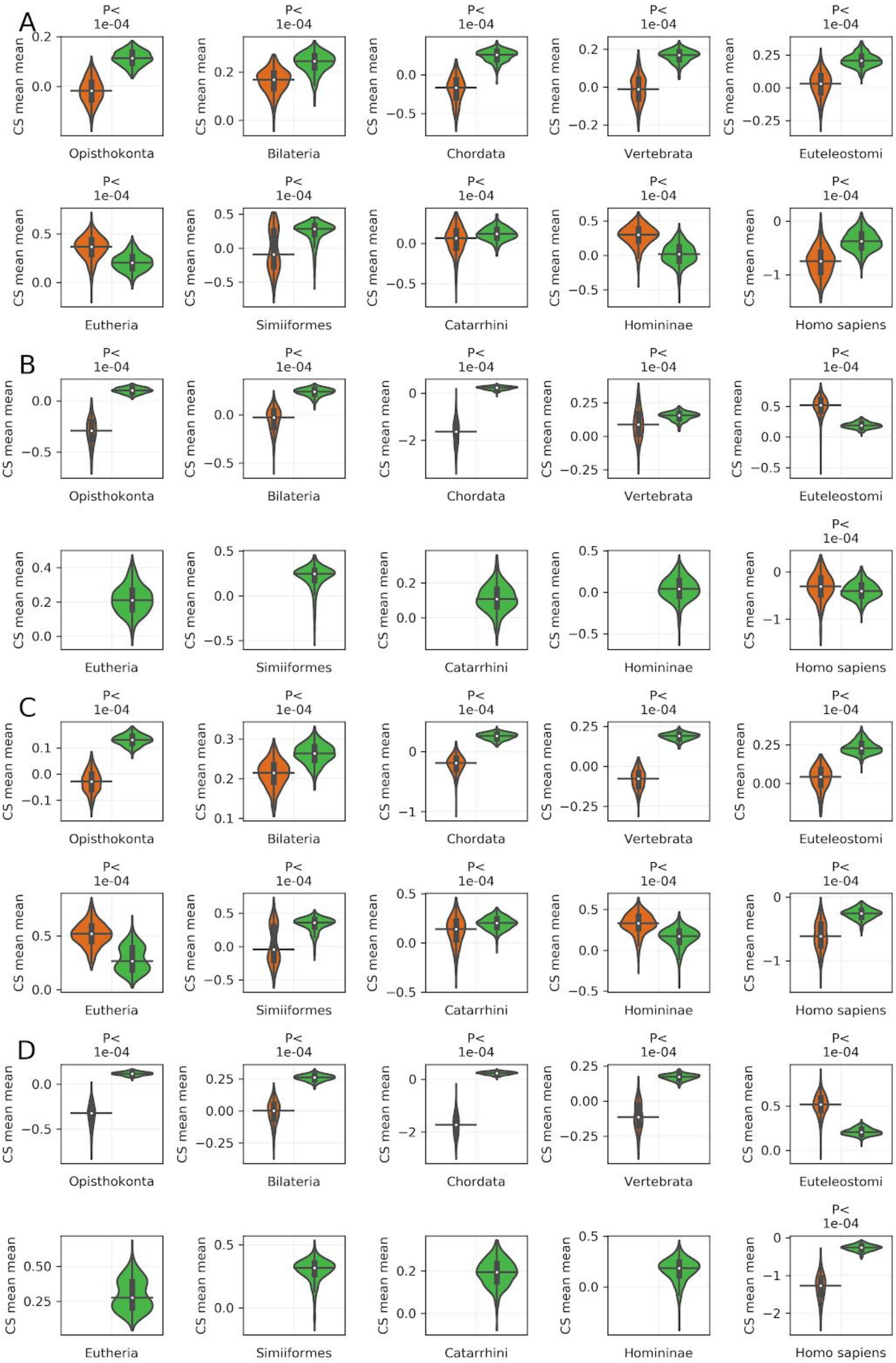

A
P<
1e-04
CS mean mean
Opisthokonta
Bilateria
Chordata
Vertebrata
Euteleostomi
Eutheria
Simiiformes
Catarrhini
Homininae
Homo sapiens
B
C
D

Appendix Fig S5: Paralogs that form heteromers tend to be more deleterious upon LOF than other paralogs, largely independently from the age of paralogs.

The effect of LOF of heteromeric paralogs and non heteromeric paralogs on cell proliferation (CS) from CS2.1 dataset is shown. On the x axis, paralogs are ordered by the age in terms of dS bins (A and B) and age groups (C). The CS values per subset defined by class of paralogs (heteromer or not) and their age group is aggregated by taking the average across cell lines. Note that while heteromers are more deleterious in most of the age groups, the reverse trend is seen for a few cases.

The classification of paralogs in Panel A is based on all interactions while that in panel B and C is based on only direct interactions.
P-values from two-sided Mann-Whitney U tests are shown. On the violin plots, the medians of the distributions is shown by a horizontal black line and quartiles are indicated by a vertical thick black line. For clarity, the upper and lower tails of the distributions are not shown.

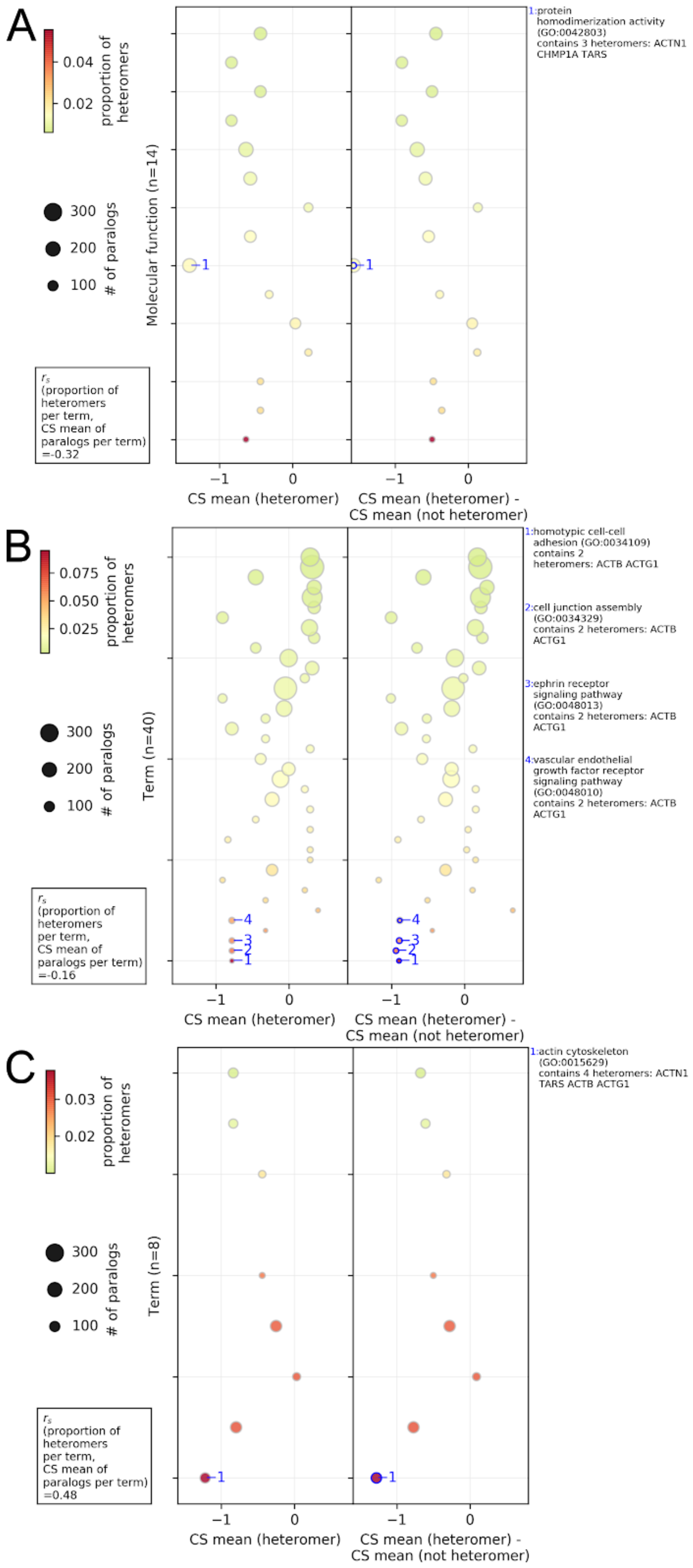
A
proportion of heteromers
0.04
0.02
# of paralogs
300
200
100
r_s (proportion of heteromers per term, CS mean of paralogs per term) =-0.32
Molecular function (n=14)
CS mean (heteromer)
CS mean (heteromer) - CS mean (not heteromer)
1:protein homodimerization activity (GO:0042803) contains 3 heteromers: ACTN1 CHMP1A TARS
B
proportion of heteromers
0.075
0.050
0.025
# of paralogs
300
200
100
r_s (proportion of heteromers per term, CS mean of paralogs per term) =-0.16
Term (n=40)
CS mean (heteromer)
CS mean (heteromer) - CS mean (not heteromer)
1:homotypic cell-cell adhesion (GO:0034109) contains 2 heteromers: ACTB ACTG1
2:cell junction assembly (GO:0034329) contains 2 heteromers: ACTB ACTG1
3:ephrin receptor signaling pathway (GO:0048013) contains 2 heteromers: ACTB ACTG1
4:vascular endothelial growth factor receptor signaling pathway (GO:0048010) contains 2 heteromers: ACTB ACTG1
C
proportion of heteromers
0.03
0.02
# of paralogs
300
200
100
r_s (proportion of heteromers per term, CS mean of paralogs per term) =0.48
Term (n=8)
CS mean (heteromer)
CS mean (heteromer) - CS mean (not heteromer)
1:actin cytoskeleton (GO:0015629) contains 4 heteromers: ACTN1 TARS ACTB ACTG1

Appendix Fig S6: Association between the GO gene sets of paralogs, their probability of heteromerization and the effect of gene LOF on cell proliferation, in case of the heteromers defined by the 'direct PPI' only.

Gene set analysis for the Molecular Functions, Biological Processes and Cellular Components aspect are shown in the panel A to C respectively.

In each panel, average CS values of paralogs (heteromer or not heteromer) belonging to a gene set were used in the analysis. GO terms are sorted according to their proportion of heteromeric paralogs (i.e. # of heteromers/ # of paralogs). The size of the circles represent the number of paralog pairs in a category and the colors represent the proportion of heteromers in the category. In the left panel, average CS value of heteromers per category is shown on the x-axis. In the right panel, the difference between the average CS value of the heteromers and average CS value of the non-heteromers is plotted in the right panel. The terms with significant difference between the average CS value of the heteromers and average CS value of the non-heteromers (estimated by two-sided t-test) are annotated with the blue edges. Descriptions of the representative significant GO terms with the highest difference are shown in the right side-panel. Spearman rank correlation between the proportion of the heteromers in the GO terms and the average CS value of paralogs in the term (r_s(# of heteromers / # of paralogs per term, CS mean of paralogs per term)) is shown in left right corner. Only GO molecular functions with more than 10% of the number of paralogs in all the gene sets are shown.

See Dataset EV4 for GO terms and annotations shown on this figure. Note that not all gene sets are independent because some genes are in several categories.

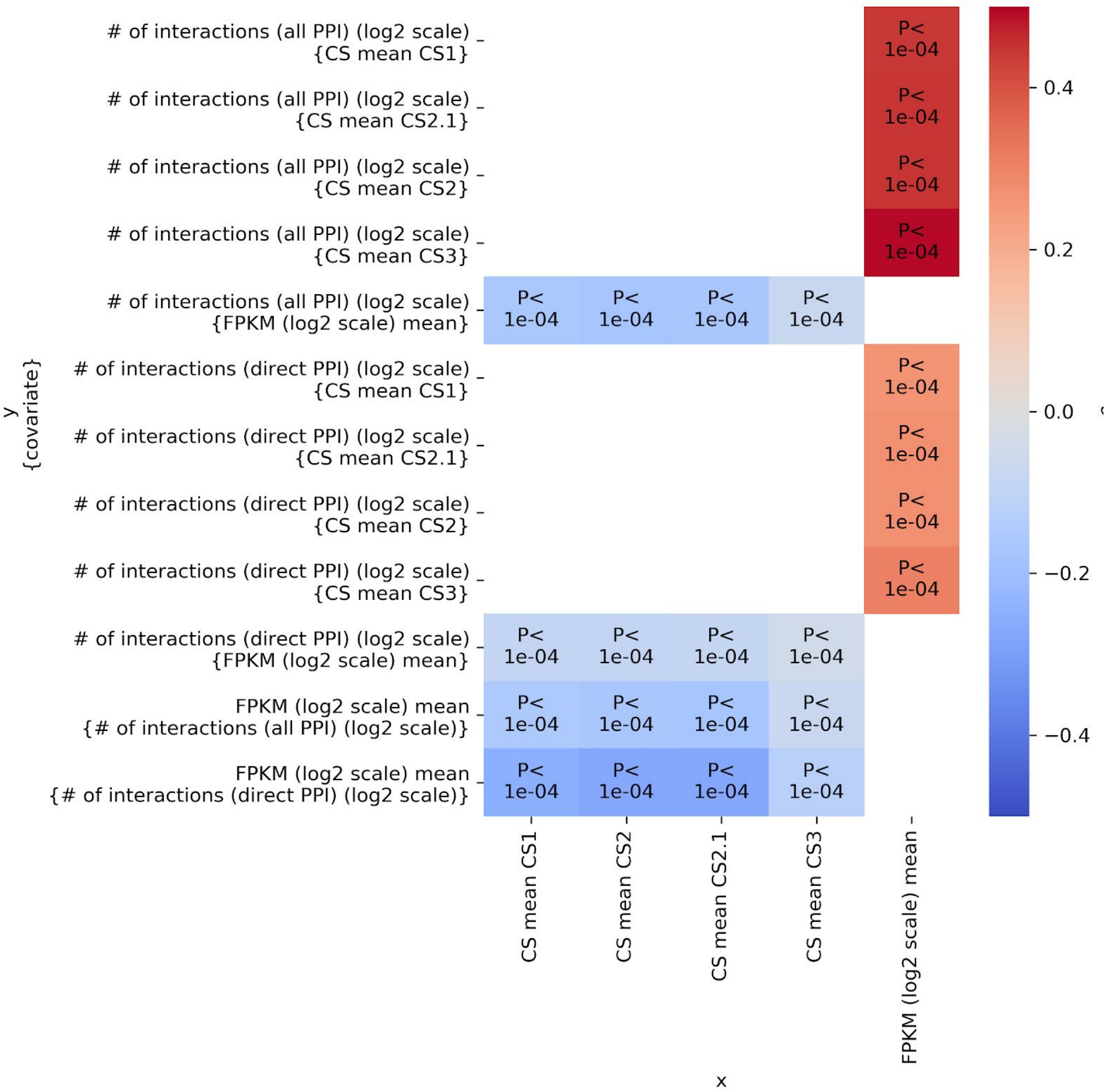

Appendix Fig S7: Correlations between the effect of LOF of a gene on cell proliferation, mRNA expression and number of protein-protein interaction partners.

Similar analysis as that of Fig 4A, but with individual CS datasets and direct PPI.

The effect of gene LOF on cell proliferation as measured in terms of CS values is correlated with mRNA expression and number of protein-protein interaction partners. Partial correlations were estimated in terms of Spearman correlation coefficients (ρ) between each pair of factors while controlling for the third factor (covariate, indicated in the curly brackets). The associated P-values are denoted on the heatmap.

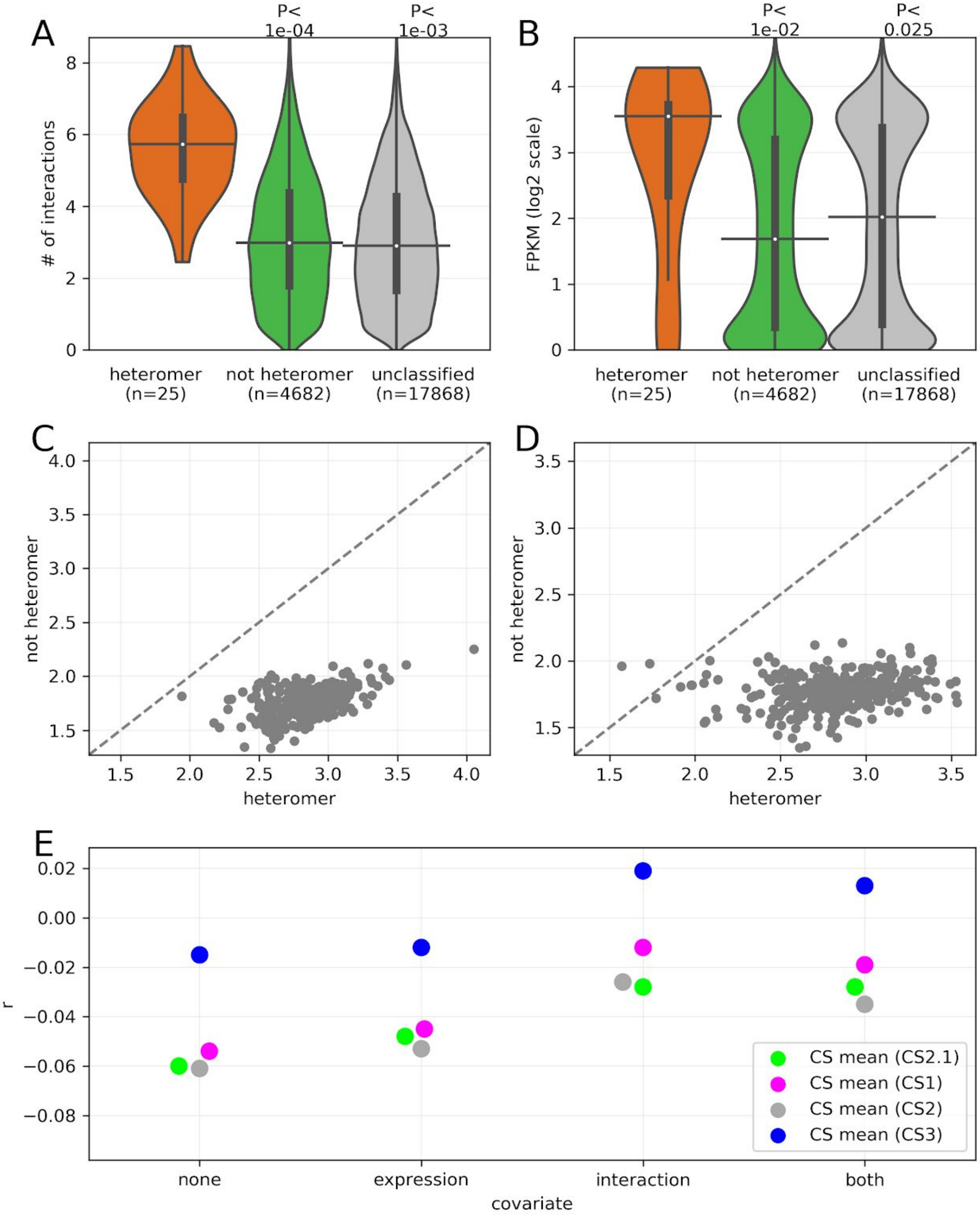

Appendix Fig S8: Relationship between the effect of LOF of a gene on cell proliferation, mRNA expression and number of protein-protein interaction partners. Related to Fig 4.

A) Similar analysis as that of Fig 4B, but for heteromers identified with ‘direct PPI’ only. Paralogs that form heteromers have more interacting partners compared to

non-heteromers. Number of interactions are in log2 scale.

B) Similar analysis as that of Fig 4C, but in case of heteromers identified with ‘direct PPI’ only. Paralogs that form heteromers show higher expression than non-heteromers.

C) Cell-line wise comparison of mRNA expression between paralogous heteromers and paralogous non-heteromers identified from ‘all PPI’ (panel **C**) and ‘direct PPI’ (panel **D**). Similar analysis as that of Fig 2B, except here for mRNA expression values. Each point represents the mean FPKM (log2 scale) score for a class (heteromer or not heteromer) in an individual cell line (n=374). In each case the ~99% of the points are separated by the diagonal (dashed gray line) indicating cell-line independent systematic effects.

E) Similar analysis as that of Fig 4D, but in case of heteromers identified with ‘direct PPI’ only. Partial correlations were determined in terms Spearman correlation coefficients (r, shown on the y axis), between CS values and a paralog status (heteromer or not, binary variable, 1 : heteromer, 0 : not heteromer). The correlations were determined while controlling for none of mRNA expression and number of interactions, only mRNA expression, only number of interactions or both (as shown on the x axis).

In panels **A** and **B,** P-values from two-sided Mann-Whitney U tests are shown. On the violin plots, the medians of the distributions are shown by a horizontal black line and quartiles by a vertical thick black line. For clarity, the upper and lower tails of the distributions are not shown.

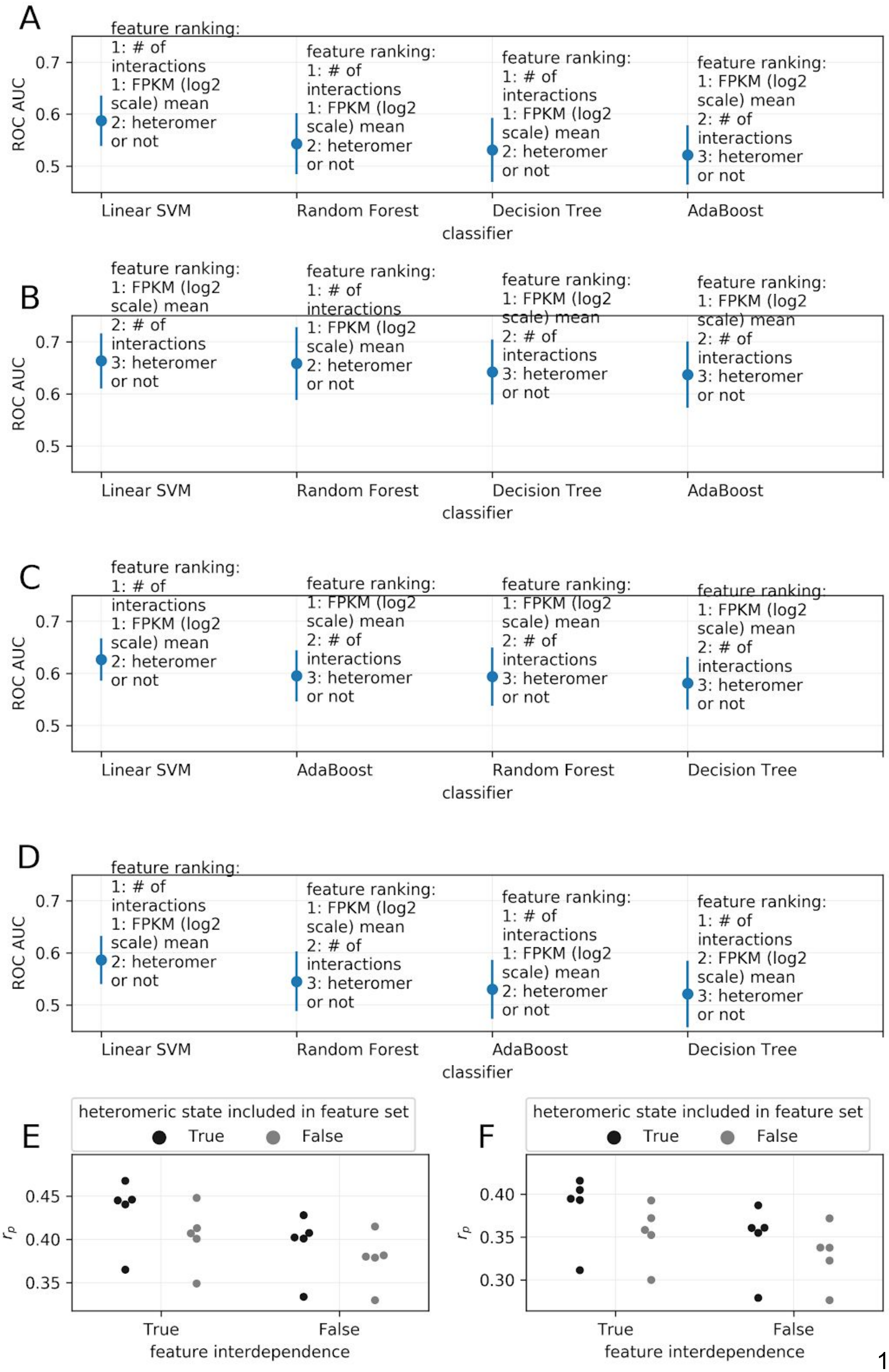
A
feature ranking: 1: # of interactions 1: FPKM (log2 scale) mean 2: heteromer or not
feature ranking: 1: # of interactions 1: FPKM (log2 scale) mean 2: heteromer or not
feature ranking: 1: # of interactions 1: FPKM (log2 scale) mean 2: heteromer or not
feature ranking: 1: FPKM (log2 scale) mean 2: # of interactions 3: heteromer or not
ROC AUC
0.7
0.6
0.5
Linear SVM
Random Forest
Decision Tree
AdaBoost
classifier
B
feature ranking: 1: FPKM (log2 scale) mean 2: # of interactions 3: heteromer or not
feature ranking: 1: # of interactions 1: FPKM (log2 scale) mean 2: heteromer or not
feature ranking: 1: FPKM (log2 scale) mean 2: # of interactions 3: heteromer or not
feature ranking: 1: FPKM (log2 scale) mean 2: # of interactions 3: heteromer or not
ROC AUC
Linear SVM
Random Forest
Decision Tree
AdaBoost
classifier
C
feature ranking: 1: # of interactions 1: FPKM (log2 scale) mean 2: heteromer or not
feature ranking: 1: FPKM (log2 scale) mean 2: # of interactions 3: heteromer or not
feature ranking: 1: FPKM (log2 scale) mean 2: # of interactions 3: heteromer or not
feature ranking: 1: FPKM (log2 scale) mean 2: # of interactions 3: heteromer or not
ROC AUC
Linear SVM
AdaBoost
Random Forest
Decision Tree
classifier
D
feature ranking: 1: # of interactions 1: FPKM (log2 scale) mean 2: heteromer or not
feature ranking: 1: FPKM (log2 scale) mean 2: # of interactions 3: heteromer or not
feature ranking: 1: # of interactions 1: FPKM (log2 scale) mean 2: heteromer or not
feature ranking: 1: # of interactions 2: FPKM (log2 scale) mean 3: heteromer or not
ROC AUC
Linear SVM
Random Forest
AdaBoost
Decision Tree
classifier
E
heteromeric state included in feature set
True
False
r_p
0.45
0.40
0.35
True
False
feature interdependence
F
heteromeric state included in feature set
True
False
r_p
0.40
0.35
0.30
True
False
feature interdependence

Appendix Fig S9: Feature importances of heteromeric state of the paralog, mRNA expression and number of PPI partners.

Shown in panel **A** to **D** is similar analysis as that of Fig 4E but with CS datasets CS1, CS2, CS2.1 and CS3 respectively. Feature importance is shown on the y axis and classification models are shown on the x axis.

E and **F)** Multiple regression analysis to predict the deleteriousness of the paralog (CS value) from feature set consisting of mRNA expression and number of PPI partners. Inclusion of heteromeric status of the paralogs in the feature set improves the regression (estimated in terms of Pearson's correlation coefficient, r_p) indicating that heteromeric status of the paralog is one of the predictors of the deleteriousness of paralogs, albeit weaker one as compared to mRNA expression and the number of PPI partners. Additionally, inclusion of interdependence in the regression (interactions of degree 2) also improves the strength of regression, indicating the interdependence between the features is of important role. Shown in panels **E** and **F** are the analyses with heteromers defined by all and direct PPIs respectively. The results of multiple linear regression are similar in the two PPI datasets used.

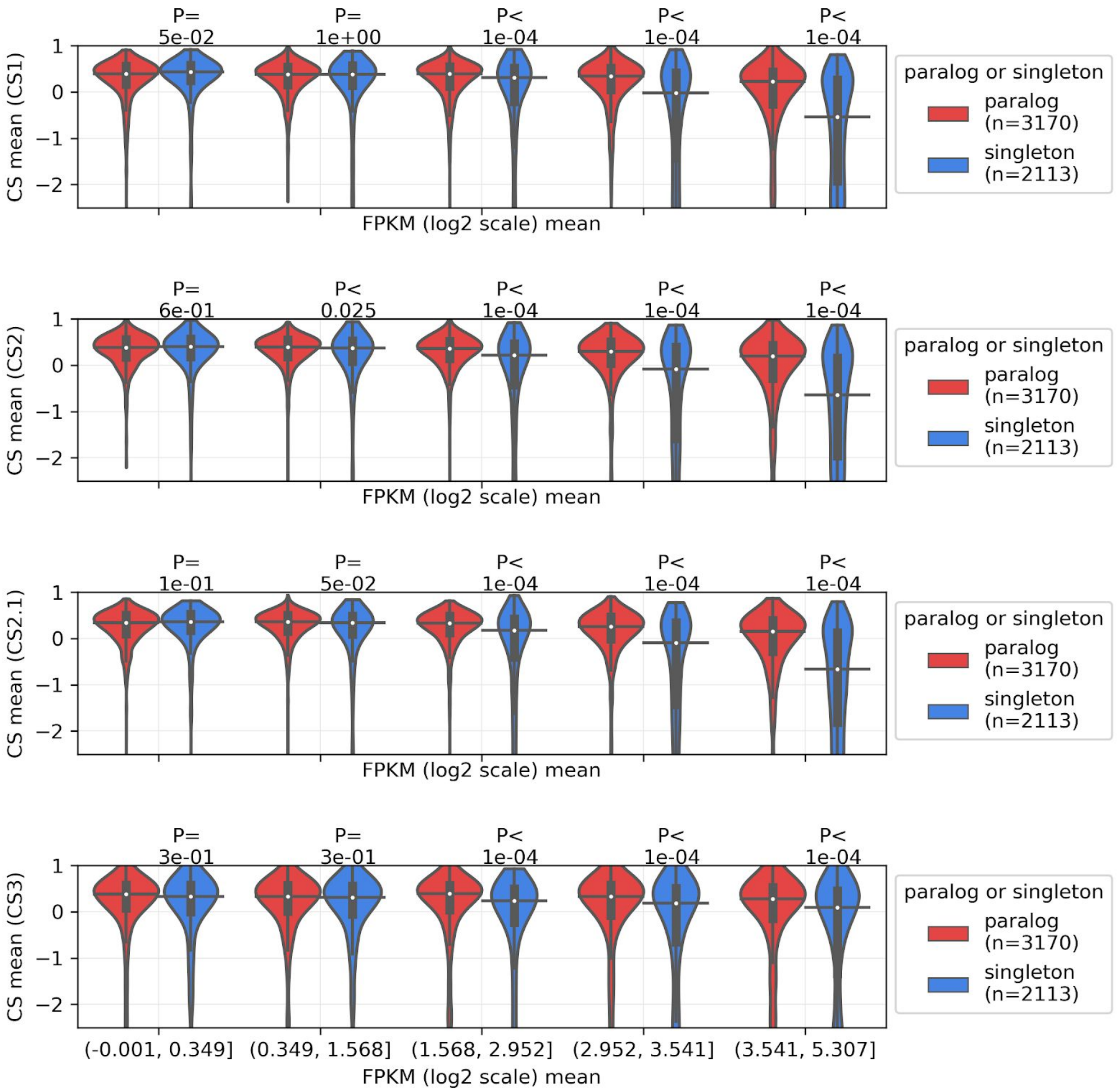

Appendix Fig S10: Dependence of the robustness of paralogs (shown on y-axis in terms of CS values) on mRNA expression (y-axis), across 4 CS datasets. Related to Fig EV3E.

mRNA expression of the genes was binned into 5 equal sized bins.

P-values from two-sided Mann-Whitney U tests are shown. On the violin plots, the medians of the distributions are shown by a horizontal black line and quartiles by a vertical thick black line. For clarity, the upper and lower tails of the distributions are not shown.

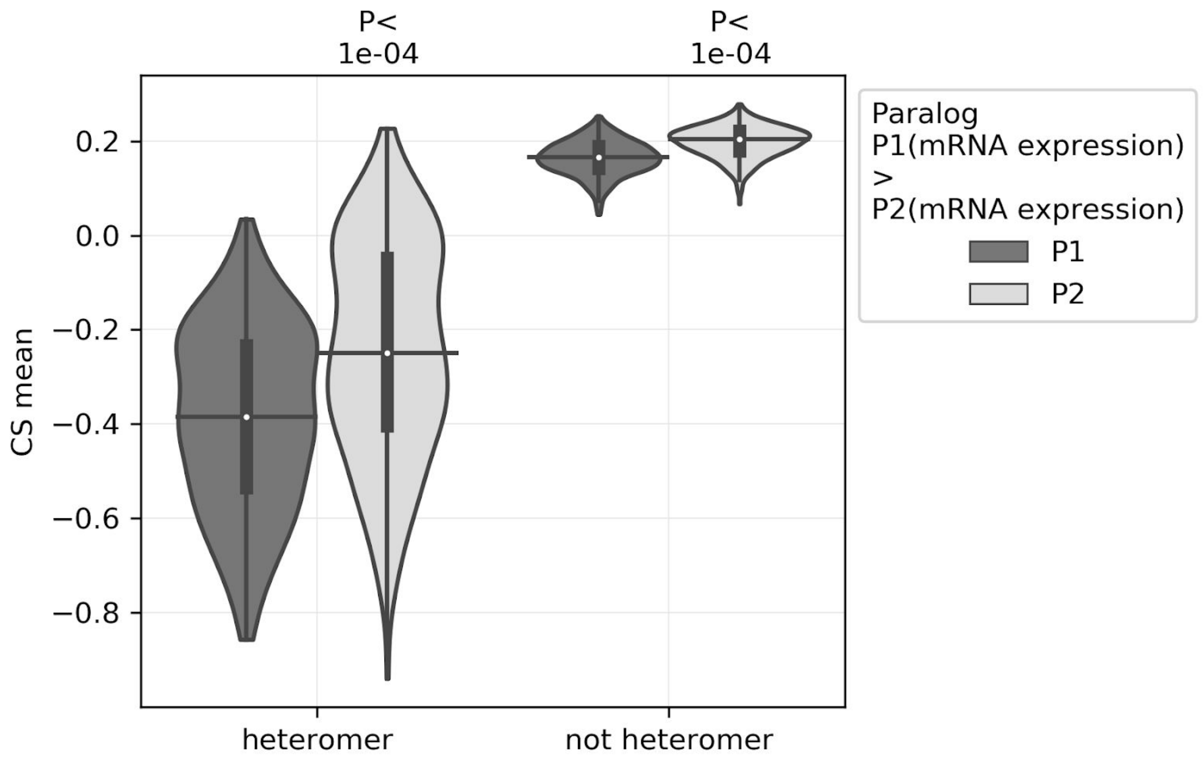

Appendix Fig S11: The most expressed paralog (P1) of a pair is more likely to be deleterious than the least expressed (P2), across 374 cell lines.

Similar analysis as that of Fig 6, but with ‘direct PPI’.

Each point represents CS value of an individual cell line.

P-values from two-sided Mann-Whitney U tests are shown. On the violin plots, the medians of the distributions are shown by a horizontal black line and quartiles by a vertical thick black line. For clarity, the upper and lower tails of the distributions are not shown.

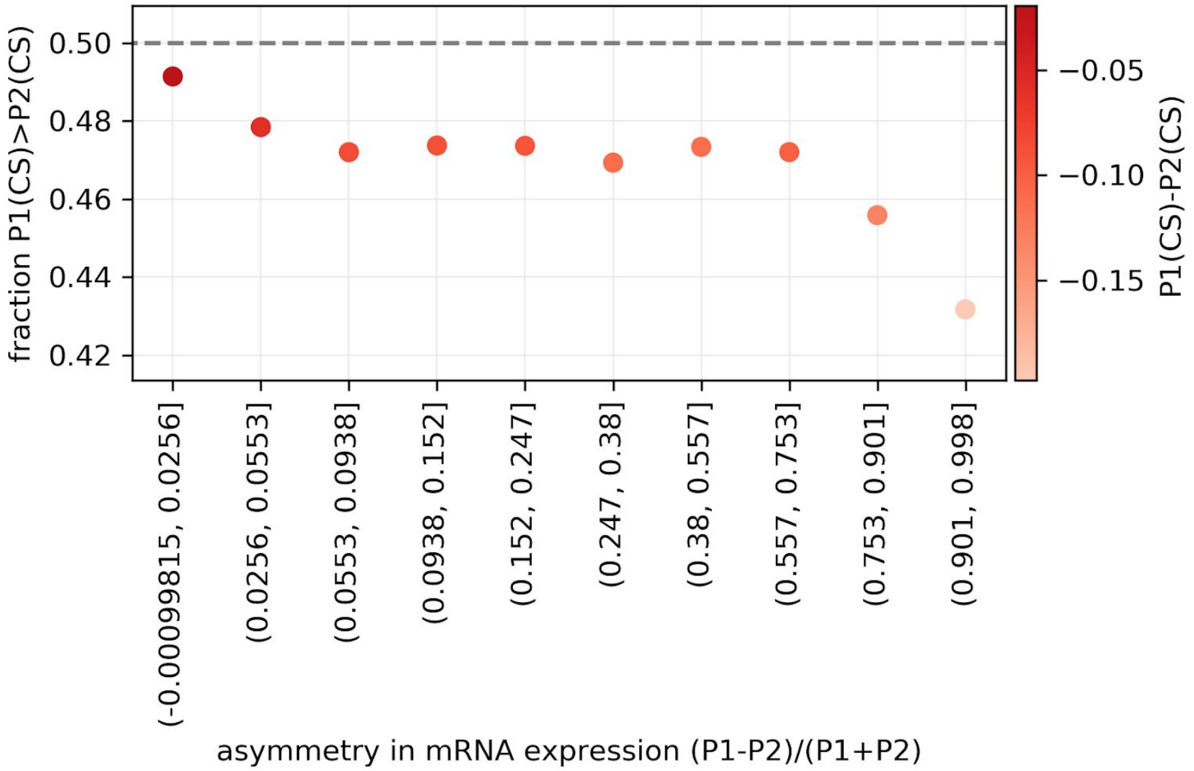

Appendix Fig S12: Relationship between the asymmetry of expression and relative deleteriousness of paralog for the CS2 dataset.
The probability that a highly expressed paralog P1 has higher CS than comparatively weakly expressed paralog P2, as a function of its normalized relative mRNA expression to P2. Similar analysis as that of Fig EV5A, but with CS2 dataset.

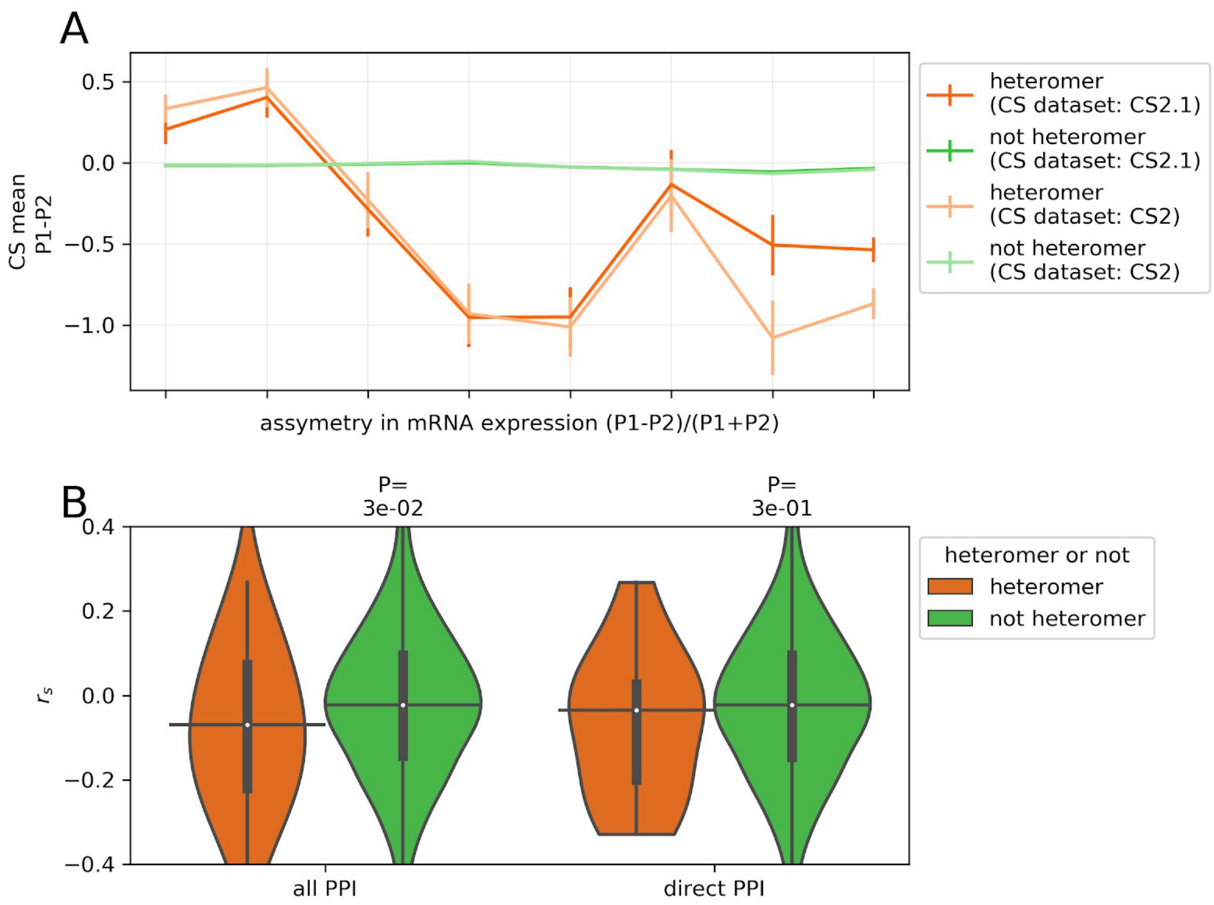

Appendix Fig S13: Relationships between asymmetry of the mRNA expression and difference in CS values. Related to Fig 6.

A) Relationship between the difference in CS of the paralog pair (P1-P2) and the asymmetry of mRNA expression levels i.e. (P1-P2)/(P1+P2), where mRNA expression of P1 is higher than P2. Values near 0 are cases in which the mRNA expression is symmetrical and asymmetrical for values near 1. The heteromers are defined by direct PPI'.

B) Average difference of CS value between P1 and P2 (P1(CS) -P2(CS)) is correlated with the asymmetry of mRNA expression (i.e. (P1-P2)/(P1+P2), where mRNA expression of P1 is greater than that of the P2), across cell lines. Similar analysis as Fig 4B, but with CS2 dataset.

P-values from two-sided Mann-Whitney U tests are shown. On the violin plots, the medians of the distributions are shown by a horizontal black line and quartiles by a vertical thick black line. For clarity, the upper and lower tails of the distributions are not shown.

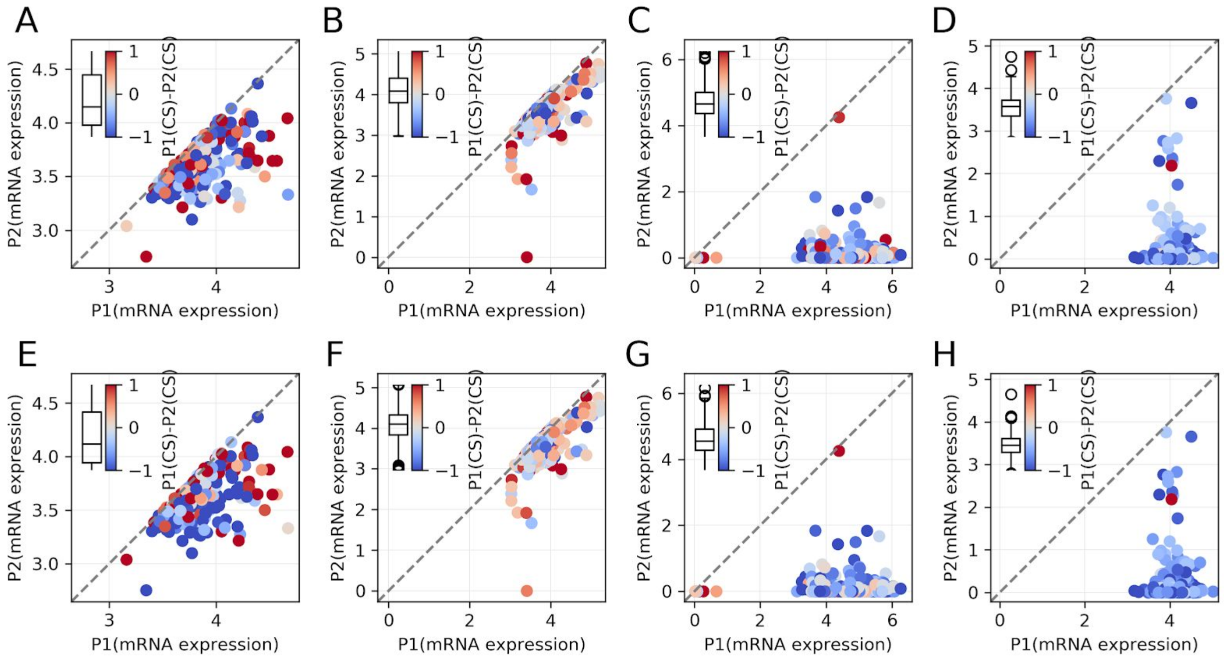

Appendix Fig S14: Relationships between the asymmetry of the mRNA expression and the difference in CS values is shown for representative heteromeric and non-heteromeric pairs of paralogs.

Each datapoint represents one cell line and is colored according to the difference of CS value for a pair. In the examples shown, the heteromers have more symmetrical expression levels than non heteromers. The results show that points away from the diagonal tend to be blue, showing that the asymmetry of expression varies per cell line and this generally correlate with the asymmetry of deleteriousness as well.

Representative heteromers: UBQLN1-UBQLN4 is shown in the 1st column of panels (panel A and E), while LMNA-LMNAB1 is shown in the 2nd column of panels (panel B and F).

Representative non-heteromers: SNX17-SNX31 is shown in the 3rd column of panels, while SPTA1-SPTAN1 is shown in the 4th column of panels.

The plots on the 1st row derive from the CS2.1 dataset, while those in the 2nd row derive from the CS2 dataset.

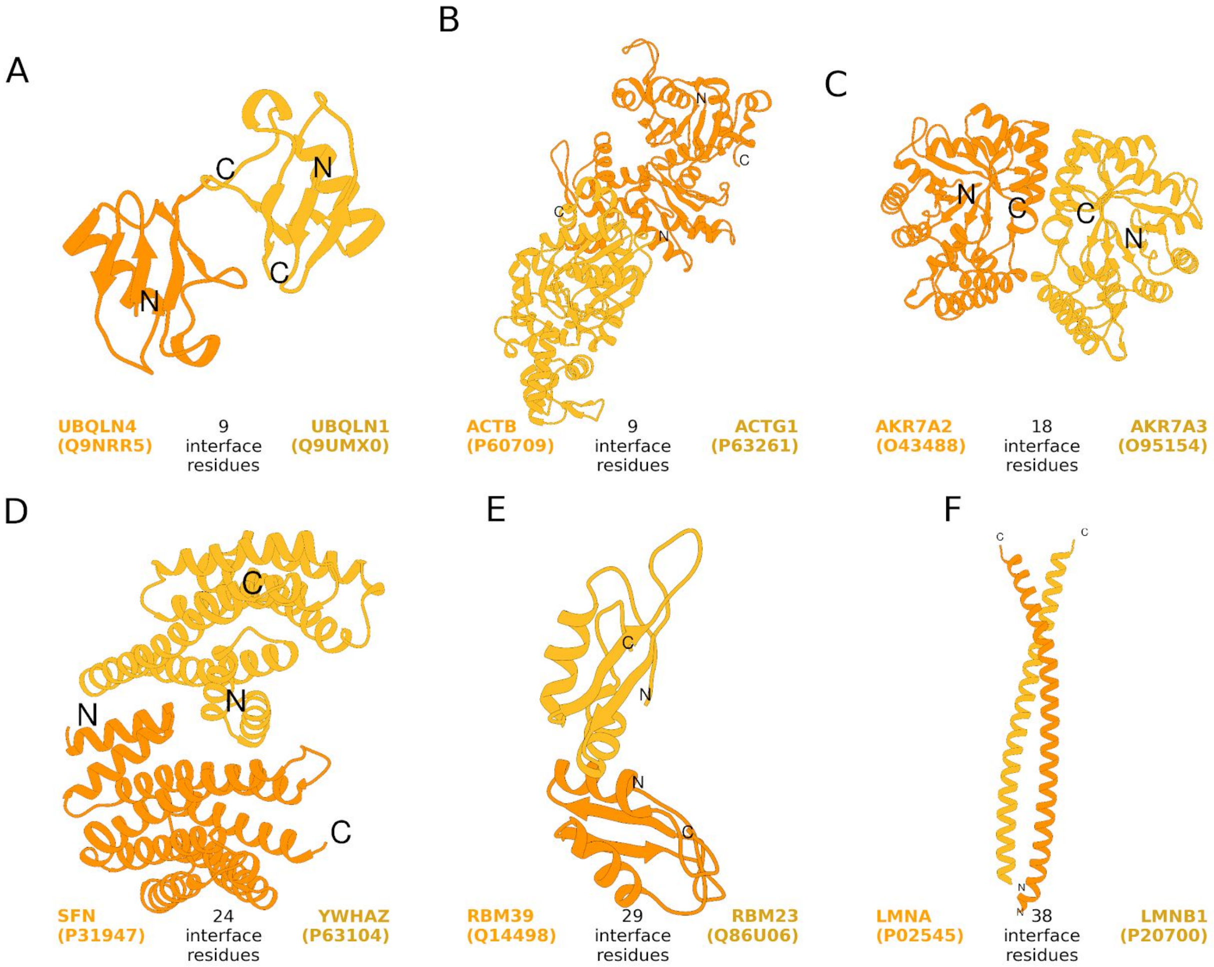

Appendix Fig S15: Structures of the representative heteromeric paralogs showing the number of interface residues.

Structures of the paralogs were obtained from Interactome3D (Mosca, Céol, and Aloy 2013) while the number of residues at the interaction interface were retrieved from Interactome INSIDER (Meyer et al. 2018). Uniprot ids of the paralogs are shown in brackets.

Appendix references

DepMap, Broad. 2018. “DepMap Achilles 18Q3 Public.” Figshare. https://doi.org/10.6084/M9.FIGSHARE.6931364.V1 [DATASET].

Meyer, Michael J., Juan Felipe Beltrán, Siqi Liang, Robert Fragoza, Aaron Rumack, Jin Liang, Xiaomu Wei, and Haiyuan Yu. 2018. “Interactome INSIDER: A Structural Interactome Browser for Genomic Studies.” *Nature Methods* 15 (2): 107–14.

Meyers, Robin M., Jordan G. Bryan, James M. McFarland, Barbara A. Weir, Ann E. Sizemore, Han Xu, Neekesh V. Dharia, et al. 2017. “Computational Correction of Copy Number Effect Improves Specificity of CRISPR–Cas9 Essentiality Screens in Cancer Cells.” *Nature Genetics* 49 (October): 1779.

Mosca, Roberto, Arnaud Céol, and Patrick Aloy. 2013. “Interactome3D: Adding Structural Details to Protein Networks.” *Nature Methods* 10 (1): 47–53.

Shifrut, Eric, Julia Carnevale, Victoria Tobin, Theodore L. Roth, Jonathan M. Woo, Christina T. Bui, P. Jonathan Li, Morgan E. Diolaiti, Alan Ashworth, and Alexander Marson. 2018. “Genome-Wide CRISPR Screens in Primary Human T Cells Reveal Key Regulators of Immune Function.” *Cell* 175 (7): 1958–71.e15.

Wang, Tim, Kıvanç Birsoy, Nicholas W. Hughes, Kevin M. Krupczak, Yorick Post, Jenny J. Wei, Eric S. Lander, and David M. Sabatini. 2015. “Identification and Characterization of Essential Genes in the Human Genome.” *Science* 350 (6264): 1096–1101.
