# Supplementary material for: Paralog dependency indirectly affects the robustness of human cells
Source: Mol Syst Biol. 2019 Sep 24;15(9):e8871. doi: 10.15252/msb.20198871 (PMC6757259; doi:10.15252/msb.20198871)
Supplement: Supplementary file 2 — Expanded View Figures PDF [file MSB-15-e8871-s002.pdf]

Expanded View Figures

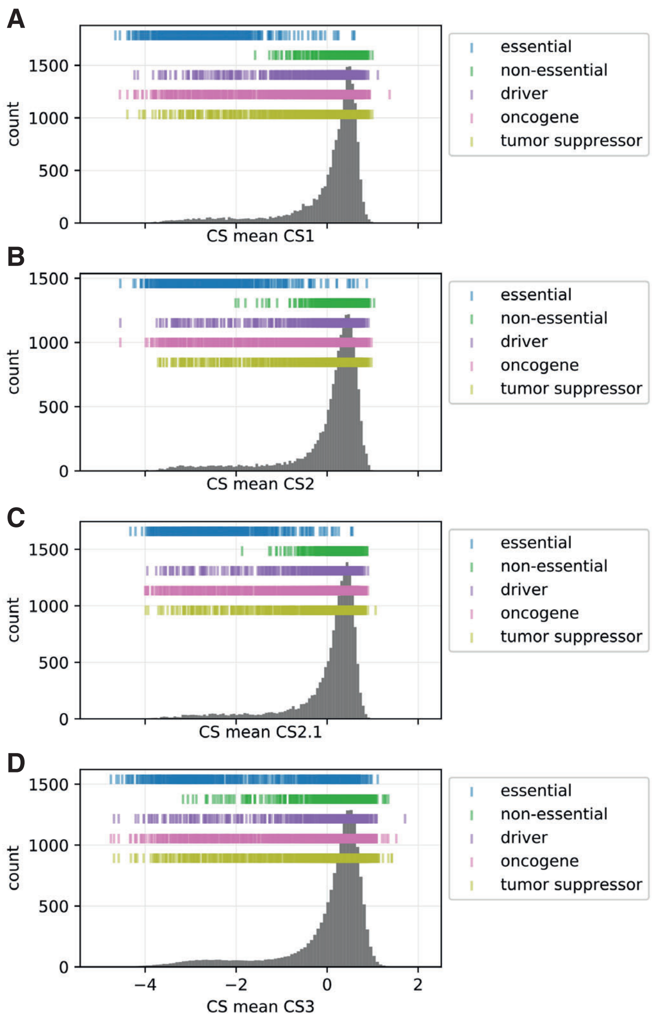

Figure EV1. Distribution of CS values in the 4 CS datasets.

A–D The locations of essential and non-essential genes [taken as a union set of genes reported by DepMap, 2018 and BAGEL (Hart & Moffat, 2016)] are denoted on the distributions. The locations of the cancer drivers, oncogenes, and tumor suppressors are also denoted on the distribution (derived from Lever *et al*, 2019).

Source data are available online for this figure.

Figure EV2. Association between the biological processes and cellular components of paralogs, their probability of heteromerization, and the effect of gene LOF on cell proliferation, in the case of the heteromers defined by the "all PPI" only.

A, B Gene set analysis for Biological Processes and Cellular Components is shown in panels (A) and (B), respectively. Average CS values (*x*-axis) of paralogs (heteromer or not heteromer) belonging to a gene set were used in the analysis. In each panel, GO terms are sorted according to their proportion of heteromeric paralogs (i.e., # of heteromers/# of paralogs). The size of the circles represents the number of paralog pairs in a category, and the colors represent the proportion of heteromers in the category. In the left panel, average CS value of heteromers per category is shown on the *x*-axis. In the right panel, the difference between the average CS value of the heteromers and average CS value of the non-heteromers is shown on the *x*-axis. The terms with significant difference between the average CS value of the heteromers and average CS value of the non-heteromers (estimated by two-sided *t*-test) are annotated with the blue edges. Descriptions of the representative significant GO terms with the highest difference are shown in the right-side panel. Spearman rank correlation between the proportion of heteromers in the GO terms and the average CS value of paralogs in the term [r_s(# of heteromers/# of paralogs per term, CS mean of paralogs per term)] is shown in the lower left corner. Only GO molecular functions with more than 10% of the number of paralogs in all the gene sets are shown. See Dataset EV4 for GO term annotations shown on this figure. Note that not all gene sets are independent because some genes are in several categories.

Source data are available online for this figure.

▶

© 2019 The Authors

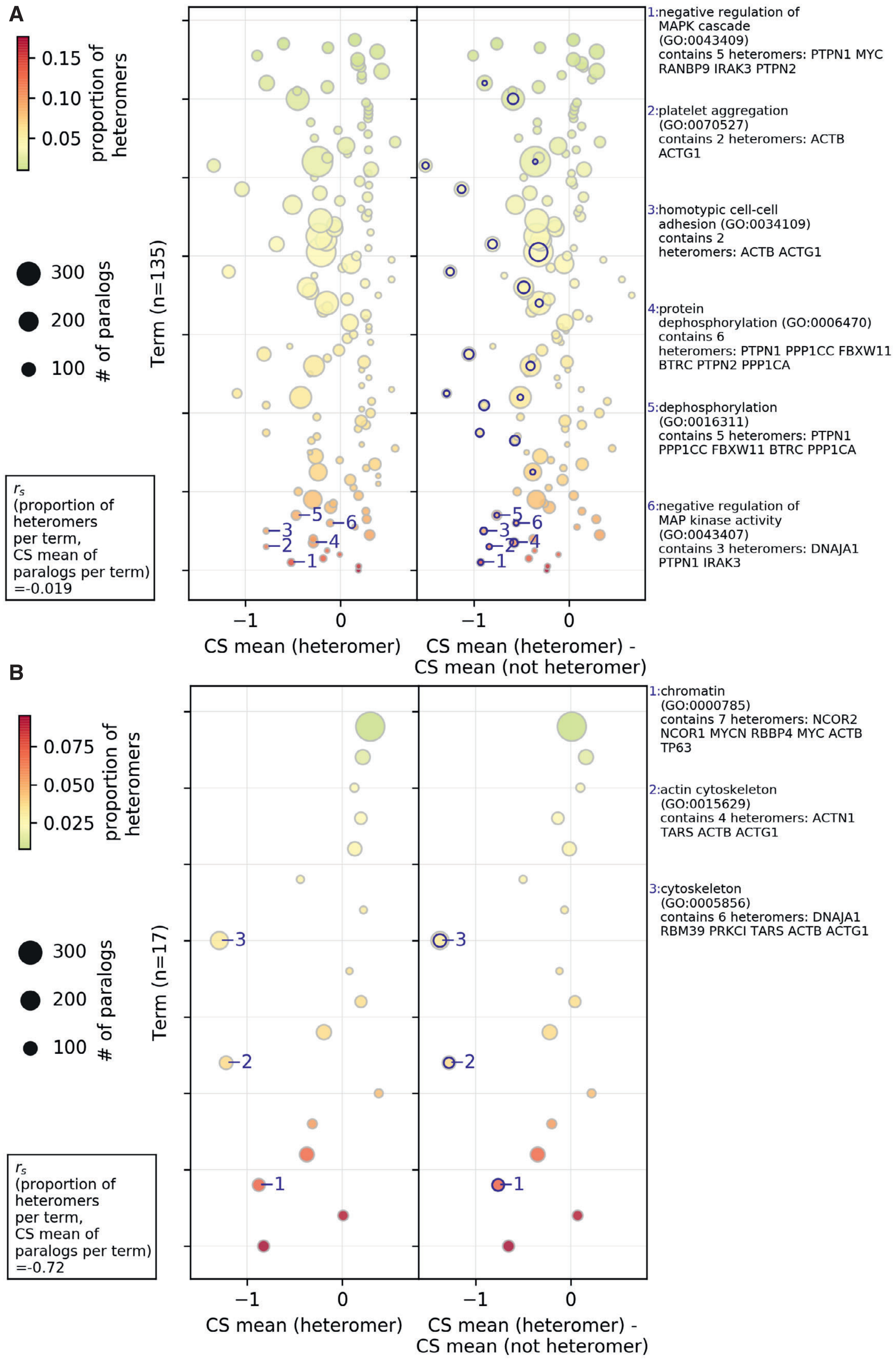

Figure EV2.

 © 2019 The Authors

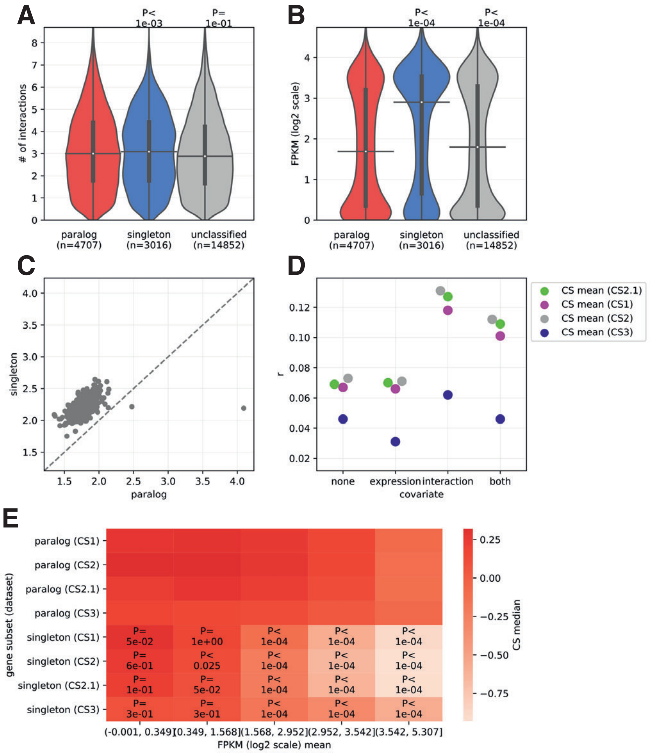

Figure EV3. Paralogs have fewer interaction partners and lower mRNA expression compared to singletons.

A Paralogs have fewer interaction partners than singletons. Number of interactions is in log_2-transformed.

B Paralogs have lower mRNA expression than singletons. mRNA expression of genes is shown in terms of log_2 of FPKM.

C Across the majority of cell lines, the average mRNA expression of paralogs is lower than that of singletons. Each point represents the average mRNA expression (FPKM in log_2 scale) for a class (paralog or singleton) in an individual cell line. All points are above the diagonal (dashed gray line), indicating that the effect is systematic and largely cell-line independent.

D Partial Spearman correlation coefficients (r, shown on the y-axis) between the CS value and a paralog status of a gene (paralog or singleton, binary variable, 1: paralog, 0: singleton). The correlations were calculated while controlling for none of mRNA expression and number of interactions ("none"), only mRNA expression ("expression"), only number of interactions ("interaction"), or both ("both") (as shown on the x-axis). Controlling for mRNA expression leads to the greater loss of correlation for interactions. The mRNA expression of paralogs is a better contributor to correlation between the CS values and the status of the gene being paralog or singleton (binary variable), than the number of interaction partners.

E Interdependence of the robustness of paralogs (shown in terms of CS score, y-axis) on the mRNA expression (on y-axis). Gene subsets, i.e., paralog or singleton and CS datasets are shown in rows. In the columns, mRNA expression of the genes binned into five equal-sized bins is shown. Median of the CS values of the genes in each subset is shown on the heatmap. The P-values from two-sided Mann–Whitney U tests for the comparison of distributions of the CS values of the paralogs versus singletons, in each CS dataset and each bin of mRNA expression, are denoted on the heatmap. Distributions of CS values in each case are shown in Appendix Fig S10.

Data information: In panels (A) and (B), P-values from two-sided Mann–Whitney U tests are shown. On the violin plots, the medians of the distributions are denoted by a horizontal black line, whereas the quartiles from the median value are indicated by a vertical thick black line. For clarity, the upper and lower tails of the distributions are not shown.

Source data are available online for this figure.

© 2019 The Authors

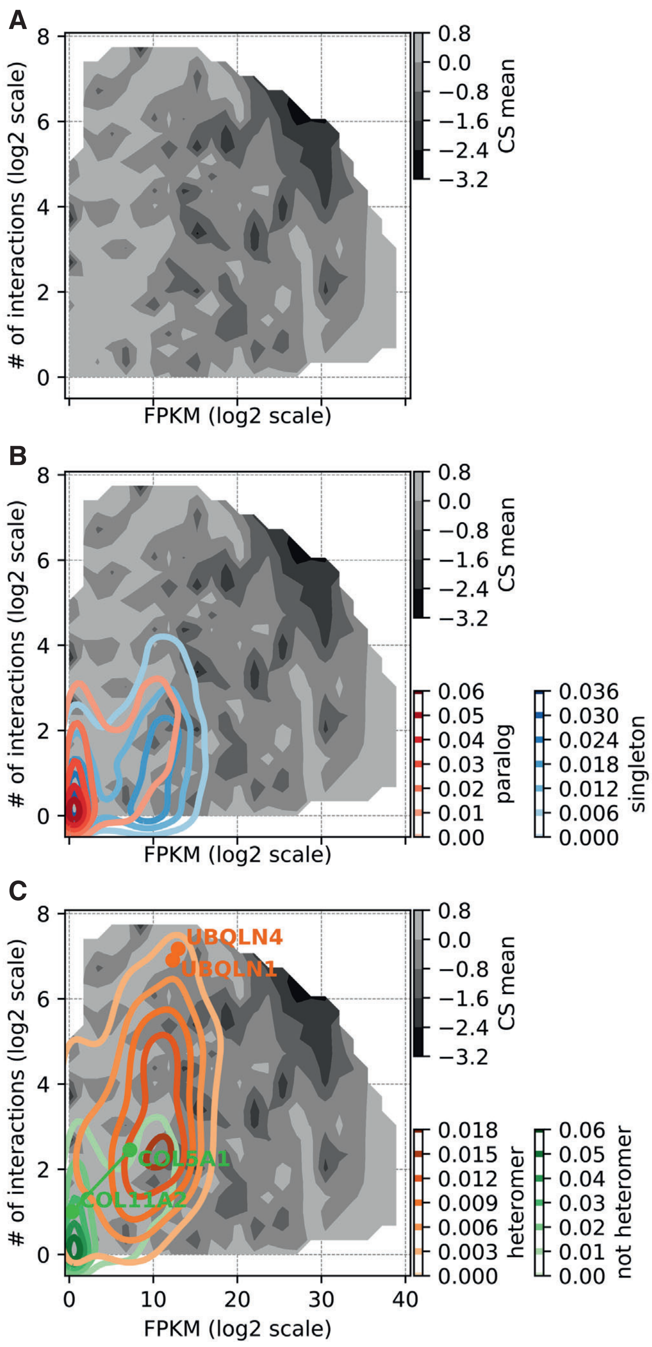

Figure EV4. Landscape of the robustness of human cell lines to LOF, considering direct physical interactions only.

A RNA expression level (log_2 scale FPKM scores) and number of direct protein–protein interaction partners (log_2 scale) are strong determinants of the deleteriousness of gene LOF. The landscape shows CS values as a function of these two parameters. Regions of the landscape with high mRNA expression and large number of interactions clearly show lower CS values.

B Kernel density estimates for paralogs and singletons are overlaid on the landscape to indicate their level of occupancy. The density of paralogs is biased strongly toward lower expression levels compared to singletons.

C Similar to (B), heteromeric paralogs and non-heteromeric ones are overlaid on the landscape to indicate their level of occupancy. The density of heteromers is biased strongly toward higher number of protein interaction partners, compared to non-heteromers. The positions of representative heteromeric and non-heteromeric pairs of paralogous genes are shown on the robustness landscape.

Source data are available online for this figure.

 © 2019 The Authors

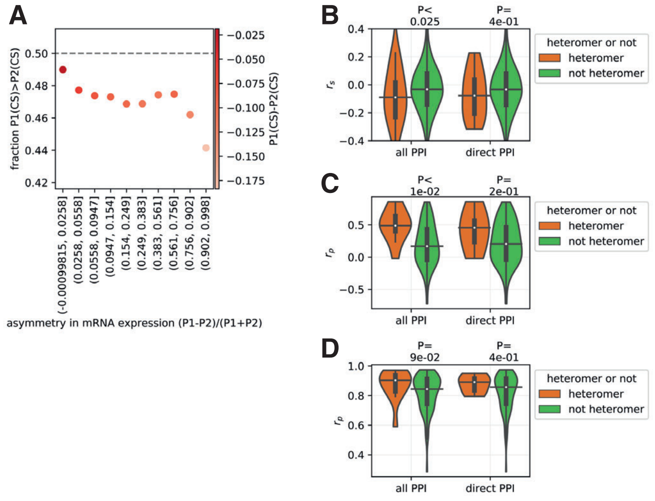

Figure EV5. Relationship between the asymmetry of expression and the relative deleteriousness of paralog.

A The probability that a highly expressed paralog P1 has higher CS than the lowly expressed paralog P2, as a function of its normalized relative mRNA expression to P2. Probability of 0.5, shown by dotted line, indicates that it is equally likely that paralog P1 would have greater CS than P2 and paralog P2 would have greater CS than P1. Probability of less than 0.5 indicates paralog P2 would have greater CS than P1. The scaled asymmetry of expression is shown on the *x*-axis. On the left, P1 is more likely to have higher CS value (less deleterious) and expression is symmetric. On the right, P1 is more likely to have relatively lower CS value (more deleterious) and expression is asymmetric. Asymmetry in mRNA expression (*x*-axis) was binned into 10 equal size bins. The color of the points represents the average difference of CS value in the bin. Similar analysis with the CS2 dataset is shown in Appendix Fig S11A.

B The average difference of CS values between P1 and P2 (P1(CS) − P2(CS)) is correlated with the asymmetry of mRNA expression [i.e., (P1 − P2)/(P1 + P2), where mRNA expression of P1 is greater than that of the P2], across cell lines. Each point in the distribution corresponds to the correlation for a single pair of paralogs. r_s: Spearman correlation coefficient. Similar analysis with CS2 dataset is shown in Appendix Fig S11B. See Appendix Fig S12 for relationships between asymmetry of the mRNA expression and difference in CS values for representative pairs of heteromers and non-heteromeric paralogs.

C Extent of the transcriptional coregulation in heteromers versus non-heteromers. mRNA expression of the paralogs was correlated across 374 cell lines. r_p: Pearsons's correlation coefficient. mRNA expression values were *z*-score normalized before estimating correlations.

D Extent of the post-transcriptional coregulation in heteromers versus non-heteromers. Protein expression of the paralogs was correlated across 49 cell lines. While estimating the partial correlation, the protein expression of the paralogs was controlled with the mRNA expression. r_p: Pearsons's correlation coefficient. Protein and mRNA expression values were *z*-score normalized before taking the correlations.

Data information: In panels (B–D), *P*-values from two-sided Mann–Whitney *U* tests are shown. On the violin plots, the medians of the distributions are shown by a horizontal black line and quartiles by a vertical thick black line. For clarity, the upper and lower tails of the distributions are not shown.

Source data are available online for this figure.
